# Adolescent Health-Risk Behavior and Community Disorder

**DOI:** 10.1371/journal.pone.0077667

**Published:** 2013-11-20

**Authors:** Sarah E. Wiehe, Mei-Po Kwan, Jeff Wilson, J. Dennis Fortenberry

**Affiliations:** 1 Department of Pediatrics, Indiana University School of Medicine, Indianapolis, Indiana, United States of America; 2 Department of Geography and Geographic Information Science, University of Illinois, Urbana-Champaign, Illinois, United States of America; 3 Department of Geography, School of Liberal Arts, Indiana University-Purdue University Indianapolis, Indianapolis, Indiana, United States of America; Tulane University, United States of America

## Abstract

**Background:**

Various forms of community disorder are associated with health outcomes but little is known about how dynamic context where an adolescent spends time relates to her health-related behaviors.

**Objective:**

Assess whether exposure to contexts associated with crime (as a marker of community disorder) correlates with self-reported health-related behaviors among adolescent girls.

**Methods:**

Girls (N = 52), aged 14–17, were recruited from a single geographic urban area and monitored for 1 week using a GPS-enabled cell phone. Adolescents completed an audio computer-assisted self-administered interview survey on substance use (cigarette, alcohol, or marijuana use) and sexual intercourse in the last 30 days. In addition to recorded home and school address, phones transmitted location data every 5 minutes (path points). Using ArcGIS, we defined community disorder as aggregated point-level Unified Crime Report data within a 200-meter Euclidian buffer from home, school and each path point. Using Stata, we analyzed how exposures to areas of higher crime prevalence differed among girls who reported each behavior or not.

**Results:**

Participants lived and spent time in areas with variable crime prevalence within 200 meters of their home, school and path points. Significant differences in exposure occurred based on home location among girls who reported any substance use or not (p 0.04) and sexual intercourse or not (p 0.01). Differences in exposure by school and path points were only significant among girls reporting any substance use or not (p 0.03 and 0.02, respectively). Exposure also varied by school/non-school day as well as time of day.

**Conclusions:**

Adolescent travel patterns are not random. Furthermore, the crime context where an adolescent spends time relates to her health-related behavior. These data may guide policy relating to crime control and inform time- and space-specific interventions to improve adolescent health.

## Introduction

Though the physical and emotional resources within homes clearly influence adolescent health and well-being, the surrounding physical area and its social milieu, loosely understood as the residential neighborhood, also plays a role. In fact, poor health outcomes cluster at various levels of area aggregation from country to census block group [1], [2]. Characteristics of these contextual areas (such as collective efficacy and poverty rates) based on or derived with various schemes of area aggregation are often observed to spatially correlate with health outcomes [3], [4]. Although past research tends to identify a general correlation between the qualities of neighborhoods where adolescents live and their health, how specific sociogeographic contexts outside their homes influence this relationship remains unclear [5].

This limited understanding of how adolescents interact with the physical and social spaces of their neighborhoods is an important barrier to the success of health promotion and disease prevention efforts. Previous studies of context primarily use arbitrary administrative areas (e.g., census tracts or block groups) or buffers surrounding participants' residential addresses to assess neighborhood contextual exposure. However, these areal units often do not fully or accurately characterize where adolescents spend time and interact with others at the micro-geographic level (e.g., spending time on the front stoop, street corners, vacant lots, or other places without adult supervision). They also cannot differentiate contextual influences that adolescents experienced at various distances from home (e.g., area immediately surrounding the home versus areas farther away from home but that are normally considered part of their residential neighborhood). As contextual influences on adolescents' health behaviors (e.g., community disorder) may vary at the micro-geographic level even within their residential neighborhoods, using arbitrary buffers or administrative units to assess their exposure seriously constrains our understanding of how specific contexts affect health outcomes [4], [6], [7]. A few studies include path data (data that capture the locations and routes taken over time) [8]–[12], but most do not examine how health behaviors vary by micro sociogeographic context within a neighborhood or by where an individual spends time.

Path data are particularly important in mobile populations where the exposure of interest occurs outside the home. Adolescence is developmentally characterized by increasing autonomy and mobility, and adolescents have a great deal of latitude to choose and use the environments where they spend time. Since adolescents spend only about half their time at home [13], environments outside the home may exert substantial influence on adolescents' health-related behaviors. In particular, behaviors such as cigarette smoking, alcohol and other drug use, and partnered sexual activity are associated with substantial morbidity in adolescence and have been linked to neighborhood and community influences, independent of those found in the home and family [14]–[17].

In order to better understand potential mechanisms by which neighborhood contexts outside but near the home might influence adolescent health-related behaviors, we asked if time spent in areas with higher prevalence of reported crimes (as an indicator of community disorder) was correlated with self-reported health-related behaviors among adolescent girls living in one area of Indianapolis, Indiana. This relatively homogenous neighborhood was chosen to specifically evaluate health-related behaviors associated with adolescent path exposures, in addition to those directly associated with areas immediately surrounding the home.

## Materials and Methods

### Ethics statement

This research was reviewed and approved by the Indiana University Institutional Review Board. We obtained written consent from each participant and her parent/guardian.

### Study design and population

The Pearl Grlz study is a prospective study of adolescent girls (N = 52), aged 14–17 years, living in one area of Indianapolis, Indiana. Girls were recruited from this area by approaching potential participants in clinic settings and in neighborhood venues as well as through flyers and announcements in the target community and a website/Facebook site. The racial-ethnic composition of our cohort reflected the larger community, with 63% self-identified Black, 31% White, and 6% Latina participants. Inclusion criteria in addition to age, gender and residence was the ability to speak and understand English.

Each participant was monitored for 4 one-week periods over the course of a year using a GPS-enabled cell phone during the study period of 2008–2011. Participants took a baseline audio computer-assisted self-administered interview (ACASI) survey indicating demographic characteristics as well as self-reported health-related behaviors. The first of the 4 one-week periods for all participants were used for this analysis.

### Quantitative data on context

Path data were collected using a global positioning system (GPS)-enabled cell phone which each participant carried during monitoring periods. The cell phone used assisted GPS signaling to determine longitude and latitude using both cell tower and satellite triangulation (accuracy: 20 feet horizontally, 36 feet vertically). The phones transmitted a device ID, timestamp, and GPS coordinates every 5 minutes to a secure server. Location was not assessed when the phone was off. In some cases, a location was identified only using cell tower triangulation when GPS satellites were not accessible by the phone. We assessed the data quality of locations identified only from cell towers by comparing the distance between cell tower locations and prior/following satellite locations. We determined that cell tower data were not reliable. Several points identified by cell tower data indicated implausible travel speeds to another location and were not always consistent with more accurate satellite locations. As a result, we interpolated data using only satellite-derived locations. For points with missing data (due to phone being off or no satellite data available, 15% of path points), location data were interpolated using the most recent satellite location under several stringent assumptions. The closest satellite location was used if the missing data period was less than 8 hours *and* data points before and after the missing period were at the same location (<30 meters apart). Straight-line imputation was used if missing data comprised less than a one hour period and data points before and after the missing period were not at the same location (>30 meters apart). Remaining missing data were excluded from analyses (<7% of path points). Sensitivity analyses were performed using less stringent criteria (100 and 200 m distances reducing missing data to 6% and 5%, respectively) with no differences in reported outcomes.

**Figure 1:**
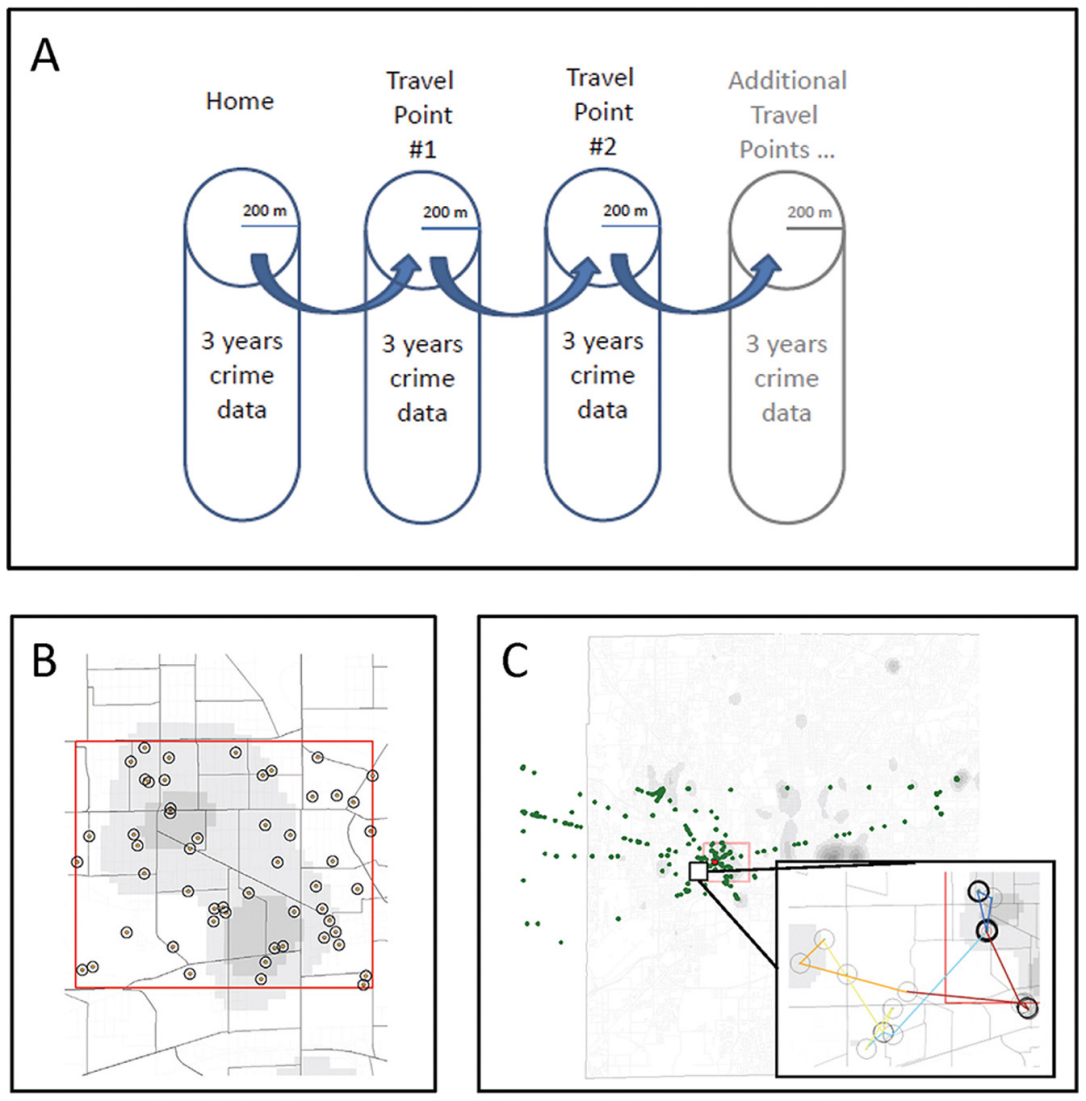
Methodology and measurement of areas of crime prevalence. (A) Conceptual depiction of path pattern exposure data to areas of crime prevalence. Each 5 minute path point includes the 3-year crime data within a 200-meter (∼1 block) buffer of this point. (B) Location of participant homes (yellow) and 200-meter buffers (black) within the study recruitment area (red) with background levels of crime ‘hot-spots’ (grey shading) and Census block groups (grey lines). (C) Path points of 1 participant (green) within and outside of the study area (red) with the inset indicating a zoomed area of path with points linked using color coding (red in the morning to blue in the evening), 200-meter buffers (black) and crime hot-spots (grey shading) and Census block groups (grey lines).

We used crime as an indicator of neighborhood disorder because it has been correlated with adverse health outcomes [18] and point-level crime data were readily available for our study area. In this study, crime was measured using geocoded locations of Unified Crime Reports (UCR) Part 1 Offenses filed in the study area. UCR Part 1 Offenses, as classified by the Federal Bureau of Investigation, include violent crime (i.e., criminal homicide, aggravated assault, robbery, forcible rape) and property crime (i.e., burglary, larceny-theft, motor vehicle theft, arson). Point-level crime data for a 3-year period (2007–2009) were aggregated in a 200-meter radius around each GPS path point (Figure 1). A 3-year period was used to provide a more stable indicator of prevalence of crime in an area. A 200-meter (approximately 1 city-block) radius was selected because we hypothesized that mobile adolescents could visually and aurally perceive the social context within this area. We used crime counts within buffers rather than crimes per capita as a proxy for social disorder based on recommendations from the criminology and crime mapping literature that caution against bias caused by the use of population-based crime rates for small areas [19]. In addition, we specifically do not state ‘crime’ as the exposure in this paper as the 3-year crime count was intended solely as an indicator of the type of area the girls were spending time in. Thus, we refer to this measure more generally as ‘community disorder.’

Geocoded crime report location data were available for Marion County, which encompasses the city of Indianapolis, Indiana (USA). Point-level crime data were not available for neighboring counties. For 200-meter buffers around girl's path points which had at least 50% of the area falling within Marion County, the total crime count within the buffer was estimated based on the available data and an assumption of uniformity. Specifically, the total number of crimes within buffers with <100% but ≥50% of their area in Marion County was estimated as: (observed crime count in known data area/area of buffer in Marion County) * total buffer area. Less than 0.01% of the total path points had buffers that overlapped the border of Marion County by <50%. Buffers around girls' path point that had >50% of their area outside of Marion County were excluded from analyses (3% of path points).

### Outcome measures

Participants completed an ACASI capturing demographic characteristics and self-reported health behaviors that correlate closely with primary causes of adult morbidity and mortality [20]. Substance use (including cigarette, alcohol and marijuana use) and sexual intercourse within the last 30 days were coded as binary variables. Questions were drawn from the CDC Youth Risk Behavior Survey which has shown good reliability for substance use questions [21]. A comparable recall period was used for sexual behavior. Questions on sensitive behaviors such as sexual activity, using ACASI and a short recall period, has been shown to have reliable recall [22]–[24].

### Analysis

We described the number and percent of participants with various demographic and behavior characteristics overall and by reported health-related behavior. We assessed the time spent at home as well as various distances from home by health-related behavior. We presented mean crime exposures within 200-meters by reported health-related behavior, using home and path data and during school and non-school days. We compared mean crime exposures between each risk group (any substance use and any sexual intercourse) and a referent group reporting neither behavior using t tests.

Contextual exposure to prevalence of crime was compared among participants reporting engaging or not engaging in each of the health-related behaviors of interest. Using Stata/MP, we created average crime counts within 200-meters of each participant's path points based on time of day and day of week (school and non-school days). Exposure by time of day is displayed in 100 increments, each increment representing 14.4 minutes of the day (e.g., 12:00–12:14am). These path points can be considered as a representative sample of all the locations a participant visited or passed through during the monitoring periods. We took the mean crime count surrounding each GPS point for participants reporting each health-related behavior: substance use or no substance use, sexual intercourse or no sexual intercourse.

## Results

Twelve percent of participants reported cigarette use, 8% alcohol use and 10% marijuana use in the last 30 days (Table 1). Seventeen percent reported sexual intercourse in the last 30 days. Demographic characteristics varied among adolescents reporting no substance use or sex, substance use but no sex, sex but no substance use, and both behaviors.

**Table 1:** Cohort characteristics.

	Total	Behaviors, last 30 days			
		no substance use	substance use	no substance use	substance use
		no sex	no sex	sex	sex
**N**	52	36	7	5	4
**Race/ethnicity**					
black	63%	64%	71%	100%	0%
white	31%	28%	29%	0%	100%
Latina	6%	8%	0%	0%	0%
**Grade in school**					
7	6%	6%	0%	20%	0%
8	21%	19%	14%	0%	75%
9	31%	33%	43%	20%	0%
10	21%	19%	29%	20%	25%
11	21%	22%	14%	40%	0%
**Behavior in last 30 days**					
cigarettes	12%		43%	0%	75%
alcohol	8%		43%	0%	25%
marijuana	10%		57%	0%	25%
sex	17%		0%	100%	100%
**Percent of time spent**					
home	54%	57%	59%	34%	42%
within 200 m, not at home	13%	12%	25%	16%	2%
between 200 m–1 km	8%	7%	2%	7%	25%
between 1 km–5 km	12%	11%	9%	17%	17%
more than 5 km away	14%	14%	5%	27%	14%

Participants spent about half their time at home during the monitoring period, and the majority of time in areas surrounding their home when not at home (Table 1). Participants who reported sexual intercourse in the last month spent less time at home; at least a quarter of their points occurred 5 kilometers or more away from home.

Prevalence of crime within 200 meters (one block) of a participant's home varied by her reported health behaviors (Table 2). Participants reporting substance use lived in areas of higher crime prevalence, compared to those reporting neither sexual intercourse nor substance use (p = 0.02). Similarly, participants who reported engaging in any sexual intercourse had greater prevalence of crime around their homes, compared to those reporting neither sexual intercourse nor substance use (p<0.001). When analyzing path locations, exposure to areas with greater prevalence of crime was significantly higher among girls reporting substance use, compared to those reporting neither behavior (p = 0.04). Though exposures were also higher among girls reporting sexual intercourse (compared to those reporting neither behavior), these differences were not statistically significant (p = 0.17).

**Table 2:** Mean and standard deviation (sd) exposure to areas of crime prevalence (3 year aggregate) by reported behavior among all participants for exposure only based on home or using all path data*.

	home			path		
	mean	sd	p	mean	sd	p
**no sexual intercourse, no substance use**	122.9	57.0	ref	119.0	50.8	ref
**any substance use**	174.5	61.6	0.02	154.6	43.8	0.04
**any sexual intercourse**	193.7	61.3	0.00	144.7	44.2	0.17

* *P values compare participants reporting either any substance use or any sexual intercourse to participants reporting no sexual intercourse or substance use. Home represents 200 meters surrounding the residential parcel.*

**Figure 2:**
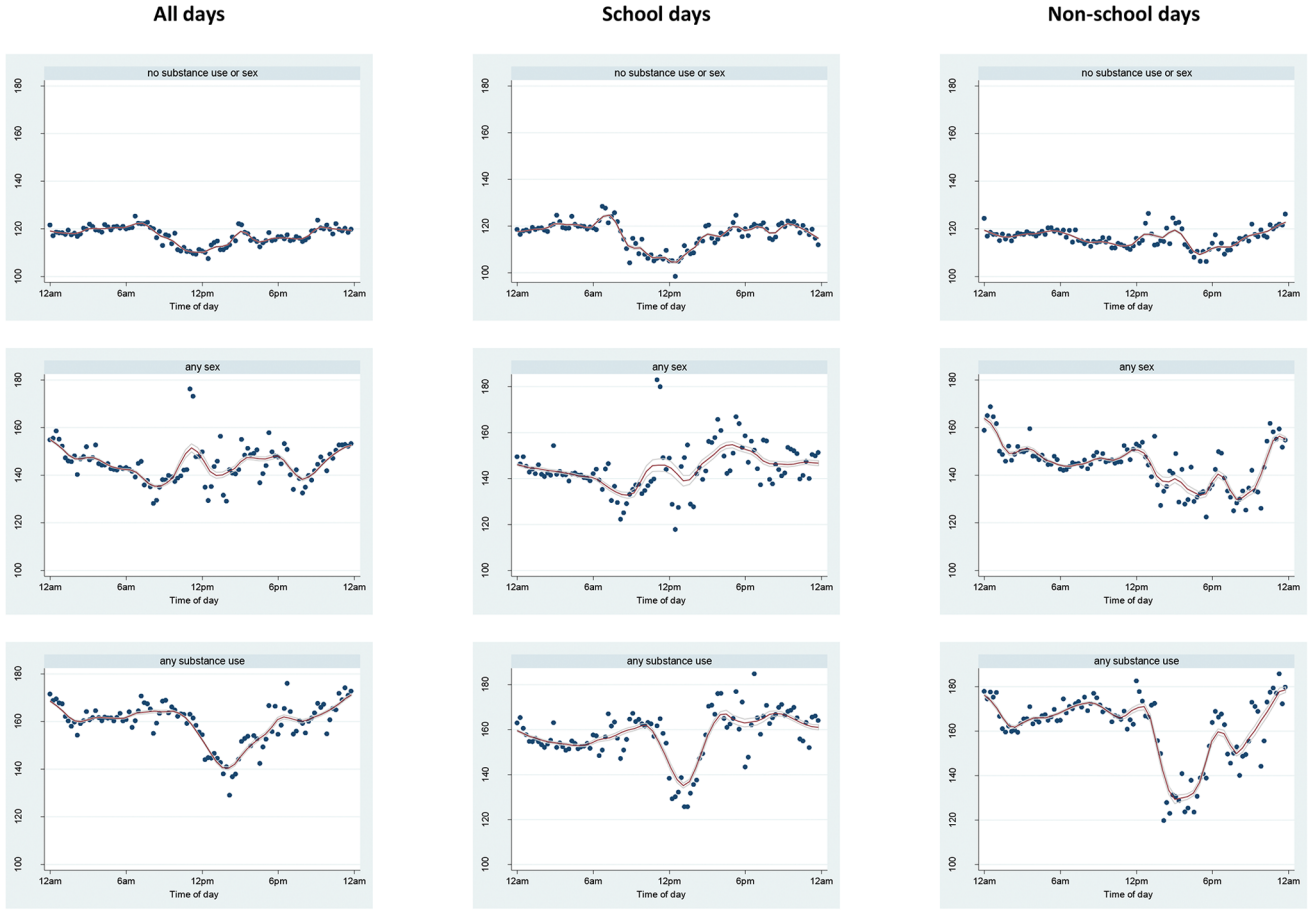
Average 200/day of week and self-reported health-related behavior in the last 30 days.

Patterns of exposure to crime prevalence varied by time of day and day of week between participants reporting no substance use or sex compared to participants reporting each of these behaviors (Figure 2). As reflected in the mean and standard deviation of exposures (Table 2), participants reporting neither substance use nor sexual intercourse had lower overall exposure to crime prevalence and this varied less by time of day and day of week than girls reporting substance use or sexual intercourse. Participants reporting substance use had the most dramatic difference by time of day, reflecting higher exposures in the evening and early morning which did not vary substantially by school and non-school days.

## Discussion

These data demonstrate clear differences in terms of *within-neighborhood* variability of adolescent women's health behaviors relating to differing exposure to the area's crime (as a marker for community disorder). Participants spent only approximately half their time at home, and substance use and sexual behaviors were significantly associated with crime characteristics of the area surrounding the home. Our adolescent participants, however, spent substantial time outside of the home, and the crime prevalence within one block of where they spent time when not at home was independently associated with their behavior. In short, participants reporting either substance use or sexual intercourse live and spend time in areas of higher crime prevalence.

Multiple studies of adolescent health behaviors consider the role of contextual influence [25]–[36]. The vast majority of studies measure contextual exposure based on a larger area and often using arbitrary administrative boundaries. A census tract, block group, or other arbitrary geographic area surrounding a residential address does not fully characterize where adolescents spend time and interact with others [37]. It is difficult to accurately assess contextual influences on individuals through using different aggregations of census tracts or other arbitrary areal units – what contextual area best represents a neighborhood, community, or significant space may influence the health outcome of interest [6], [7]. In this study, we hypothesized that a relatively small area of contextual exposure would be relevant – within a 1-block radius – and that crime as a proxy for community disorder might be a relevant contextual measure. Given adolescent girls reporting substance use lived and spent time in areas of highest crime prevalence, perhaps this represents increased access to illicit substances or increased exposure to social norms accepting substance use among minors. The relationship was weaker among girls reporting sex in the last 30 days and might indicate that crime as an indicator of community disorder is not the most salient measure of contextual influence on adolescents' sexual behavior. A different measure relating to sexual risk such as teen pregnancy prevalence may be more relevant. Adolescents reporting risk behaviors spent time in areas with lower crime prevalence when not at home which may highlight the lack of specificity or relevance of this measure or the fact that these adolescents are in fact at less risk than those who are not able to leave home. In a larger sample, it would be interesting to make this direct comparison. Regardless, this is one of the first studies to assess within-neighborhood contextual exposure and indicate relative differences in reported health-risk behaviors.

There is little understanding of the mechanisms by which neighborhoods affect health. In an article entitled “Putting People into Place,” [4] Barbara Entwisle outlines criticisms of the conceptualization and measurement of neighborhoods and their health effects. These include: (1) theories that “neighborhoods are exogenous and predetermined, and individuals are passive recipients of their effects,” (2) narrow characterization of neighborhoods with two thirds of studies focusing on measures of poverty and the remaining generally incorporating only one or two characteristics, and (3) reliance on cross sectional analyses with little attention to change within neighborhoods over time or lagged effects. Using methods and analyses similar to those in this study could start to address some of the concerns raised in her first point. Future studies incorporating a longitudinal design and multiple measures may help to further identify mechanisms and perhaps uncover causal associations between context and health.

Many studies do not report smaller area variability due to the large area of contextual measure aggregation. For many contextual measures, only aggregated data are available due to confidentiality risk, sampling methodology, or other reasons. Even when point-level data are available, they are often aggregated to census block group or tract for unclear reasons. In this study, we purposefully recruited from the same geographic area to assess within-neighborhood variability in both residential and path-related exposures. Given the significance of our findings with even a small sample, additional studies should consider smaller areas of exposure in the future.

Evidence suggests that a narrower view of context may increase our understanding of its relationship with health. In the Moving to Opportunity (MTO) program in which families in public housing were randomized to either stay in public housing, move to another location of their choice, or a low-poverty neighborhood, 5-year outcomes were mixed with respect to adolescent health behaviors [38]. One interesting finding was that girls were less likely to smoke in the intervention groups than girls in the control group, whereas boys in the intervention groups were more likely to smoke than their control counterparts. In a follow-up qualitative study in 1 of the 5 sites, researchers found that the gender differences were not due to variability in *general* where girls and boys generally spent time but rather where girls and boys *specifically* spent time [39]. Girls in intervention groups were more likely to spend time closer to home, on the front stoop, for example. Boys were more likely to congregate on street corners, parks, vacant lots, and other places without adult supervision. This suggests that within neighborhoods, and perhaps even between neighborhoods, specific areas where (and specific times when) individuals experienced contextual influences are volitionally chosen or somehow determined in a non-random way. Reasons for these choices, however, are not well understood.

This study has several limitations. First, this is a small sample of a single area of Indianapolis. We purposefully sampled in one area due to our interest in within-neighborhood variation but further study is needed to assess whether similar associations are present in other areas. Second, our analysis was limited by the availability of point-level crime report data, which was only available within Marion County. However, we employed a straightforward, conservative method to estimate crime counts in buffers with <100% but ≥50% of their area within the study area and excluded observations exceeding this threshold. Overall, this affected a very small proportion of the total path points and we have no reason to believe that there is significant difference in crime patterns across county boundaries that would be manifested within the relatively small buffer size we used. Third, this is a cross-sectional analysis in which we did not control for potentially confounding variables. These variables might include individual (such as school failure, self-esteem), family (such as family conflict, parent connectedness) and neighborhood characteristics (such as alcohol or drug availability, poverty, neighborhood norms) known to be associated with substance use and sex [36], [40]–[42]. Likewise, this study cannot differentiate whether these contexts contribute to girls' behavior choices or whether girls engaging in particular behaviors seek different environments. Given our interests, however, in primarily identifying associations for the purposes of future interventions, this study shows promise that similar analyses may identify adolescent girls at times and places where these behaviors are more likely to occur. Again, further longitudinal study is needed to assess causal relationships and, specifically, if these behaviors of interest are in fact occurring when they are in areas of high crime.

In sum, this analysis of space-time patterns for adolescent health behaviors is particularly important given the strong and consistent association between social context and health outcomes. The micro-sociogeographic context where adolescents spend time while away from home influence their health-related behaviors. Moreover, there is variability *within* neighborhoods in terms of exposure to community disorder and reported behaviors. Thus, more specific measurements of contextual exposures and individual-level analyses are warranted. In addition, there may be differences in the relationship between community disorder with either substance use or sex, indicating the complexity of the person-environment interaction with respect to each behavior. Our hope is that this within-neighborhood and path-specific contextual data collection and analysis may better inform future crime-control policy and time- and space-specific interventions to improve adolescent health. For instance, we may be able to use GPS technology to identify times and places where various health-risk behaviors are likely to occur (based on their association with more micro-measures of context such as community disorder) in order to better target health messages or other health-promoting interventions. Adolescents may be more receptive and responsive to these space- and time-specific interventions, though this assertion merits further study.
